# Semi-Quantitative, Duplexed qPCR Assay for the Detection of *Leishmania* spp. Using Bisulphite Conversion Technology

**DOI:** 10.3390/tropicalmed4040135

**Published:** 2019-11-01

**Authors:** Ineka Gow, Douglas Millar, John Ellis, John Melki, Damien Stark

**Affiliations:** 1 School of Life Sciences, University of Technology, Sydney, NSW 2007, Australia; john.ellis@uts.edu.au; 2 Genetic Signatures Ltd., Sydney, NSW 2042, Australia; doug@geneticsignatures.com (D.M.); john@geneticsignatures.com (J.M.); 3 Microbiology Department, St. Vincent’s Hospital, Sydney, NSW 2010, Australia; damien.stark@svha.org.au

**Keywords:** leishmaniasis, qPCR, bisulphite

## Abstract

Leishmaniasis is caused by the flagellated protozoan *Leishmania,* and is a neglected tropical disease (NTD), as defined by the World Health Organisation (WHO). Bisulphite conversion technology converts all genomic material to a simplified form during the lysis step of the nucleic acid extraction process, and increases the efficiency of multiplex quantitative polymerase chain reaction (qPCR) reactions. Through utilization of qPCR real-time probes, in conjunction with bisulphite conversion, a new duplex assay targeting the 18S rDNA gene region was designed to detect all *Leishmania* species. The assay was validated against previously extracted DNA, from seven quantitated DNA and cell standards for pan-*Leishmania* analytical sensitivity data, and 67 cutaneous clinical samples for cutaneous clinical sensitivity data. Specificity was evaluated by testing 76 negative clinical samples and 43 bacterial, viral, protozoan and fungal species. The assay was also trialed in a side-by-side experiment against a conventional PCR (cPCR), based on the Internal transcribed spacer region 1 (ITS1 region). Ninety-seven percent of specimens from patients that previously tested positive for *Leishmania* were positive for *Leishmania spp.* with the bisulphite conversion assay, and a limit of detection (LOD) of 10 copies per PCR was achieved, while the LOD of the ITS1 methodology was 10 cells/1000 genomic copies per PCR. This method of rapid, accurate and simple detection of *Leishmania* can lead to improved diagnosis, treatment and public health outcomes.

## 1. Introduction

Leishmaniasis is an infection caused by some species of *Leishmania* parasites that affect the skin, organs and mucosal regions of the body, leading to serious morbidity and possibly death. It is classed as a Neglected Tropical Disease (NTD), affecting 12 million people worldwide, with a further 350 million people at risk of contracting the disease [1]. With the advent of increased human international travel, due to work, tourism or war, leishmaniasis is now an emerging infectious disease, with an increased impact on global mortality and morbidity [2]. It is becoming increasingly clear that there is a need for accurate and rapid detection of *Leishmania* in the form of a standardized and validated assay, to aid diagnosis, treatment and surveillance programs. 

Many validated molecular *Leishmania* detection assays use conventional PCR (cPCR) for the detection of *Leishmania* infection [3,4,5]. Conventional PCR is a diagnostic method where DNA is amplified using a thermal cycler, amplicons are separated due to molecular weight by electrophoresis, and detected by stain (usually ethidium bromide or gel red) and UV light (via a transilluminator) [6]. This approach requires significantly more hands-on time, has a greater risk of contamination and makes multiplexing analysis more difficult if products are similar in size, compared to real-time PCR. Probe-based qPCR can overcome these issues. Additionally, specificity can be increased, and it allows for continuous monitoring of the PCR. The 18S rRNA gene (18S rDNA) is a highly conserved gene across all *Leishmania* species, despite having diverged from other similarly related species during the period Paleogene or Paleocene [7]. The gene exists in between 50–200 copies per *Leishmania* genome, making it an excellent choice for a pan-*Leishmania* detection assay [6]. To assess whether this target can be used in a novel diagnostic assay, based on bisulphite conversion and real-time PCR technologies, a series of experiments were performed to assess the limit of detection and sensitivity of the assay, and the new assay was compared to a cPCR, based on the ITS1 region, developed by Schönian et al. [8].

The development of this novel bisulphite-converted, qPCR assay methodology, based on genus-specific primer and probe designs for the 18S rDNA, and its validation, is described in this paper. Furthermore, the bisulphite conversion and purification of protozoan DNA are discussed. The assay’s limit of detection was 10 cellular or genomic copies/PCR, with clinical sensitivity and specificity demonstrated to be 97.0% and 100%, respectively. The assay takes under 2.5 hours to complete, making the assay a potential diagnostic tool for both diagnostic and research laboratories worldwide.

## 2. Materials and Methods 

### 2.1. Specimens Tested

DNA was purified from cell-cultured promastigotes of the following species: *L. donovani* (MHOM/IN/80/DD8 supplied at 2.3 × 10^7^ cells/mL), *L. braziliensis* (MHOM/BR/75/M2903 supplied at 1.63 × 10^8^ cells/mL), *L. tropica* (MHOM/SU/74/K27 supplied at 1.03 × 10^7^ cells/mL), *L. amazonensis* (MHOM/BR/73/M2269 supplied at 9.9 × 10^6^ cells/mL), *L. mexicana* (MHOM/BZ/82/BEL21 supplied at 1.51 × 10^8^ cells/mL) and *L. major* (MHOM/SU/73/5-ASKH supplied at 7.1 × 10^6^ cells/mL), obtained from the American type culture collection (ATCC, Manassas, USA). *Leishmania infantum* genomic DNA (supplied at 1.2 × 10^7^ copies/mL) was obtained from Vircell (Vircell, Granada, Spain). The assay was initially evaluated by performing a 10-fold serial dilution series of the DNA from these strains to assess the limit of detection. In addition, DNA from 67 previously extracted cutaneous clinical samples (derived from 66 unique patients), that were previously identified by St. Vincent’s Hospital, Sydney as positive for *Leishmania* by the cPCR method, during the period 2007–2016, were included in the study [8,9,10]. All DNA was initially extracted using the EZ1 tissue kit on the EZ1 biorobot (Qiagen, Hilden, Germany), in accordance with manufacturers’ recommendations regarding direct sample or following culture. Specificity was assessed by extracting DNA using standard methods from 76 negative tissue samples, previously characterised at St. Vincent’s Hospital, Sydney, and 43 potential cross-reacting organisms, and testing them in the assay (Table 1). The clinical specimens were tested in accordance with St Vincent’s Hospital ethics approval, HREC number LNR/16/SVH/231.

### 2.2. DNA Conversion and Quality Control

Genomic DNA was bisulphite converted by adding 2,880,000 or 28,800 copies of DNA/cellular standards, depending on available starting concentration, to a total volume of 150 μL with molecular grade H_2_O, then adding 250 μL 3M sodium bisulphite. Alternatively, 5 μL of DNA, previously extracted from cutaneous clinical sample DNA, were added to 145 μL of molecular grade H_2_O, then 250 μL 3M sodium bisulphite was added. One negative process control of 150 μL molecular grade H_2_O was included in each run, to check for contamination. A total of 5 × 10^5^copies/μL Lambda DNA (strain cI857 ind 1 Sam 7) (New England Biolabs, Ipswich, USA), an *Escherichia coli* bacteriophage, was added to each of these reactions, then the samples were mixed by vortexing, and incubated at 95 °C for 15 minutes. Subsequently 200 μL of this lysate was purified on the GS-mini (Genetic Signatures Ltd., Sydney, Australia) with the Sample Processing Pathogens A kit (Genetic Signatures Ltd., Sydney, Australia), according to the manufacturers’ recommendations. The eluted DNA was then diluted in molecular grade H_2_O in 10-fold dilution series, to 0.1 copy per PCR. The limit of detection (LOD) for this study was defined as the lowest concentration of DNA at which the assay detected 10 out of 10 replicates, in accordance with CLSI standards, which define the LOD as the lowest dilution where 95% of replicates are positive [11]. Cell and DNA concentrations were provided by the suppliers and copy number was calculated (https://www.thermofisher.com/au/en/home/brands/thermo-scientific/molecular-biology/molecular-biology-learning-center/molecular-biology-resource-library/thermo-scientific-web-tools/dna-copy-number-calculator.html). The GS-mini employs a closed cartridge-based system, whereby nucleic acid is bound to magnetic beads, with subsequent washing and, finally, elution steps, using heating and shaking to increase nucleic acid yield. Separate PCR areas were used for mastermix preparation, DNA seeding and PCR reactions, to prevent the possibility of PCR contamination. The addition of lambda bacteriophage DNA to the PCR reaction was used to monitor the efficiency of the bisulphite conversion, purification, and in assessing for possible false negatives due to PCR inhibition. A negative process control (molecular grade H_2_O) controlled for possible PCR contamination. 

An external positive control was developed by creating a geneblock—a synthetic double stranded 1000bp-long fragment of the 18S rDNA of *L. donovani*, (GenBank accession CP022642 positions 1047751 to 1048750), consisting of adenine, thymine, cytosine or guanine residues only. This was bisulphite converted and diluted to five copies/μL in molecular grade H_2_O, using the previously described protocol.

### 2.3. PCR Primer and Probe Design

For the 18S rDNA assay forward, reverse primers and a probe were designed, based on multiple sequence alignments of the 18S rDNA in bisulphite converted form (that is, with all cytosines converted to thymines), of the species *Leishmania aethiopica, Leishmania amazonensis, Leishmania braziliensis, Leishmania colombiensis, Leishmania donovani, Leishmania guyanensis, Leishmania infantum, Leishmania lainsoni, Leishmania major, Leishmania mexicana, Leishmania naiffi, Leishmania panamensis, Leishmania shawi* and *Leishmania tropica.* This resulted in the identification of primers PL-18S-F2 (TTATTGTTTTGGTTTTTG) and PL-18S-R2 (AAACCAAAATTACAATAAAA) and probe PL-18S-P2 (GGAGATTATGGAGTTGTGTGATA), which amplify and detect DNA fragments of 82bp in length. The exogenous control was targeted by primers Lambda New F1 (AATATTGGTAGATTATGTTTGTG), Lambda New R1 (CTATCATCAAATCATACAATACC) and probe Lambda New P1 (TGATGTGATAGGAAGAATTTGTTGTTGTTGTTGTTG), which amplify a 100bp fragment of the Lambda bacteriophage DNA. The 18S rDNA and Lambda probes are intercalating, self-quenching probes, labeled with individual fluorophores (FAM and HEX, respectively) enabling the PCR to be performed as a duplex reaction.

### 2.4. PCR Preparation, Conditions, and Interpretation

The PCR mixture was prepared by using 10μL of 2x SensiFast (Bioline), 90ng of each primer PL-18S-F2/PL-18S-R2 and 8 pmol probe PL-18S-P2, 4ng primer Lambda New F1, 40ng primer Lambda New R1 and 3pmol probe Lambda New P1, 3.5μL of template, and molecular grade H_2_O, to a final volume of 20μl. All DNA templates were tested in 10 PCR replicates. A negative template control reaction was included in each PCR run. PCRs were run on the MIC PCR thermal cycler (Bio Molecular Systems, Upper Coomera, Australia) using the following parameters: 95 °C for 3 min, and 50 cycles of 95 °C for 2 s and 50 °C for 10 s, 55 °C for 10 s (data acquisition step) and 60 °C for 10 s. 

The new assay was tested against the Schönian method by processing the equivalent concentration of *Leishmania* cells or genomic DNA, diluting these in molecular grade H_2_O, and heating at 70 °C for 15 minutes. Next, these lysates were processed on the GS-mini, using the MagPurix Viral/Pathogen Nucleic Acids Extraction Kit (Zinexts Life Science, Taipei, Taiwan) on the GS-mini, following the manufacturers’ instructions. The eluates were diluted in the same fashion as the bisulphite-treated eluates and amplified in cPCR triplicates, according to the methodology developed by Schönian et al*.,* with primers LITSR: CTGGATCATTTTCCGATG and L5.8S: TGATACCACTTATCGCACTT (targeting the ssu rRNA and 5.8S rRNA, respectively) [8].

## 3. Results 

### 3.1. Specificity of the Real-Time PCR Assay Using Quantitated Cultured Cell or Purified DNA Standards

DNA converted from the panel of seven *Leishmania* quantitated standards (*L. donovani, L. braziliensis, L. tropica, L. amazonensis, L. major, L. mexicana* and *L. infantum)* were detected by the 18S rDNA assay (Table 2). As displayed in Table 2, there was a concordance between these results and the Schönian method, as all *Leishmania* species tested were detected [8].

### 3.2. Specificity of the Real-Time PCR Assay Using Negative Control Samples

DNA, extracted from 76 negative clinical samples, did not produce any PCR products using the new assay, giving a specificity of 100% [8].

### 3.3. Specificity of the Real-Time PCR Assay Using Cross-Reactivity Specimens

To further investigate the specificity of the assay, a panel of DNA from 43 other phylogenetically related organisms, or those with a differential diagnosis related to leishmaniasis, was tested (Table 1). No PCR products were detected from any of these specimens, giving a specificity of 100%.

### 3.4. Limit of Detection of the Real-Time PCR Assay Using Quantitated Standards

**Figure 1:**
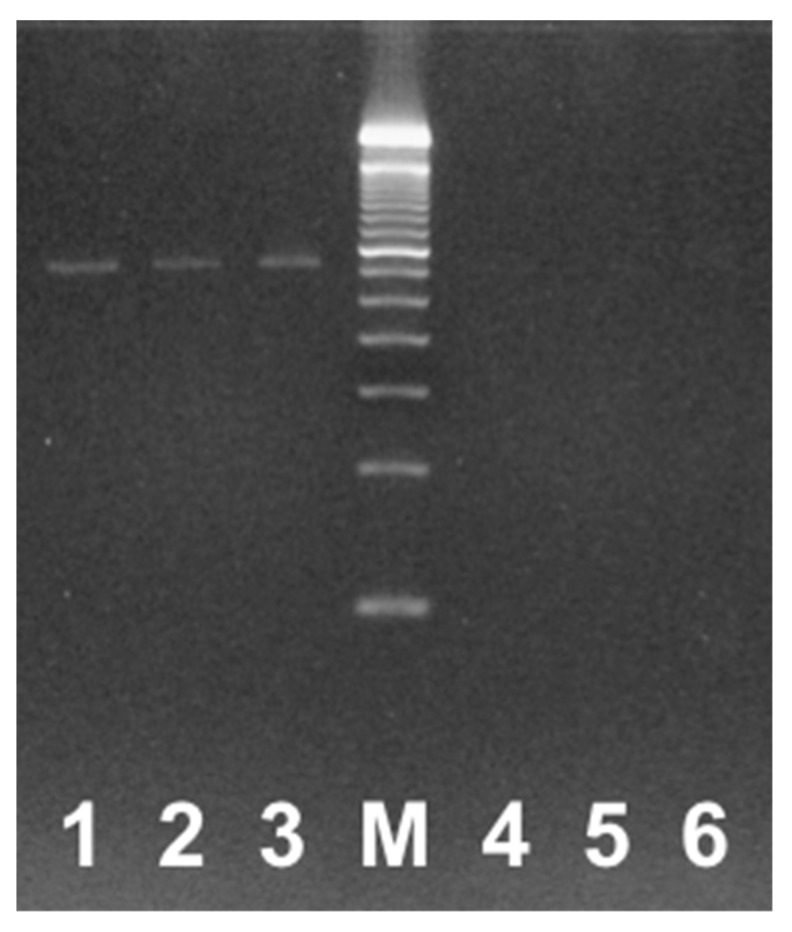
Sensitivity of the ITS1 cPCR assay for *L. braziliensis,* gel electrophoresis of conventional PCR result, using the Schönian method. Lanes 1, 2 and 3 are 100 copies/PCR; lane M is the 100bp ladder size standard, lanes 4, 5 and 6 are 10 copies/PCR.

The analytical sensitivity of the assay was evaluated using quantitated DNA and cell culture standards. Ten-fold serial dilutions were tested in the assay, and the LOD for *Leishmania* was shown to be 10 cellular/genomic copies per PCR reaction, although this LOD differed between species, as outlined in Table 2. For *L. braziliensis,* for example, the LOD was 10 cellular copies, and an average of 38.7 cycle threshold (C_T_) value was determined after testing the sample in 10 PCR replicates. When *L. braziliensis* was tested by the Schönian method, the LOD was 100 cellular copies/PCR when tested in triplicate (Figure 1). ATCC quantitation was given in cells/μL and Vircell quantitation was given in copies/μL, so this nomenclature has been upheld.

### 3.5. Sensitivity of the Real-Time PCR Assay Using Clinical Sample DNA

Previously extracted DNA from 67 clinical tissue samples was available from patients with confirmed diagnosis of cutaneous leishmaniasis. Although clinical data were not available for all specimens, of those samples with data available, 32 (72.7%) were male, and the age range was between one and 73 years. Forty-two patients had data available on previous travel; 21 (50.0%) of these patients had been to the middle east, 11 (26.2%) had been to South America, three (7.1%) had been to southern Europe, one (2.4%) to South Asia and five patients (11.9%) had been to multiple geographic regions. Reason for travel data were available for 39 patients; 26 (66.7%) were travellers, nine were immigrants (28.2%) and two (5.1%) were members of the army. Resulting cPCR (Schönian method) and restriction fragment length polymorphism analysis were used for detection and species differentiation, respectively [8]. Of the 67 clinical samples, the novel assay was detected in 65, thus 97.0 % concordance was achieved between the previous method and the 18S assay.

### 3.6. Precision of the Real-Time PCR Assay Using Quantitated Standards

**Figure 2:**
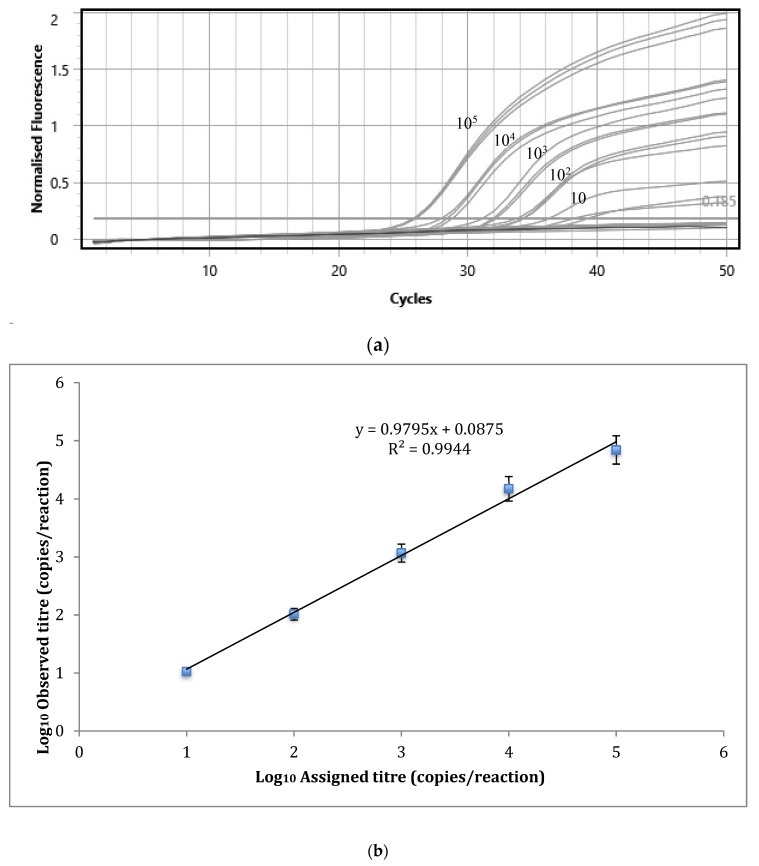
Sensitivity of the novel qPCR assay for *L. braziliensis.* (**a**) FAM channel amplification curves, using 10-fold serial dilutions from 10^5^ to 0.1 copies per PCR, tested in PCR triplicate. (**b**) Graphic depiction of the linear range of detection (10 to 10^5^ copies per reaction). Error bars represent 95% CI.

Standard curves were produced for *L. braziliensis* and *L. tropica*, testing 10-fold serial dilutions in PCR triplicates, giving an R^2^ value of 0.9945 for *L. braziliensis* (Figure 2a,b). This is a measure of the linearity of the generated curves and reflects efficiency and reproducibility. Error bars depict 95% confidence intervals, based upon two experimental replicates, comprising three PCR technical replicates each. To measure the intra-experiment precision and agreement between experiments, five experiments were each performed over five consecutive days, with three replicates at two cellular concentrations for each species (Table 3). The results considered over 236 replicates, with four negative replicates for *L. tropica* excluded. Low coefficients of variation (CVs), related to intra-experiment variability, were observed, all <10%. These findings provide additional support that this novel, real-time PCR provides efficient and precise quantification of DNA within and between experiments. 

### 3.7. Internal Control Reaction

No samples showed inhibition of the exogenous control.

## 4. Discussion

The development of a multiplexed, real-time PCR, targeting the 18S rDNA to detect all *Leishmania* species and the associated automated bisulphite conversion system, is described. The assay was validated on DNA and cell standards and the limit of detection, using seven individual strains of *Leishmania*. The LOD was compared with the method of Schönian et al. [8] and, as can be seen from Table 2, the LOD of all seven species improved upon using the real-time PCR method [8]. No cross-reactivity was observed using a panel of 43 possible cross-reacting organisms (Table 1) and 76 negative tissue samples, making the assay exclusive to *Leishmania* DNA detection. For clinical performance, the DNA of 67 previously described positive tissue samples were tested, alongside a conventional PCR method, as described by Schönian et al., and 65 samples tested positive in the 18S rDNA real-time PCR assay [8]. The Schönian method is based on the ITS1 region, a gene also located on the ribosomal DNA array, and thus present in the same number of copies as the 18S rRNA gene [12]. The novel assay includes an exogenous control, which controls for extraction and PCR performance, and an external positive control, controlling for PCR performance. The inclusion of quality controls, both internal and external, was highlighted as important in *Leishmania* detection assays [13]. The turn-around time is less than 2.5 hours from sample to result, and the system has a small laboratory footprint (the area required in the laboratory for instrumentation) of 75cm by 75cm.

The assay is based on the gene coding for the small subunit rRNA, a highly conserved region of the ribosomal DNA, located on chromosome 27. This gene was used for the detection of *Leishmania* in other assays, due to its excellent sensitivity, attributed to the fact that it is a multicopy gene, which is transcribed into abundant rRNA found in the cytoplasm, where it is predicted to be present at 10^4^ copies [7,14,15,16].

The test utilises bisulphite conversion technology, whereby the genome is simplified to three nucleobases: A, T and G (Figure S1). This simplification of the genome enables easier design of primers and probes across subtypes and species variants, as single oligonucleotide sets can be designed to cover a diverse population and reduce the need for multiplexing, in order to capture all species. Furthermore, the increase in homology allows for different primer and probe sets of differing targets to be designed with similar melting temperatures (Tm), reducing potential issues with specificity (Table S1). This was previously demonstrated in two clinical trials, where nearly 100% sensitivity and specificity were achieved by the increased homology and similar Tm of the primers and probes designed for the assays [17,18]. Bisulphite conversion technology is already in use in various diagnostic laboratories in the detection of clinical sample types, including gastro-intestinal infections [18,19]. The bisulphite conversion is included in the initial lysis step and therefore requires no extra steps by the end user. This is the first *Leishmania* detection assay exploiting bisulphite technology. The bisulphite conversion technology can also be used in an assay designed to differentiate *Leishmania* species. As there are over 20 *Leishmania* species pathogenic to humans, these will need to be multiplexed with up to four other targets into at least five panels [20]. A similar Tm greatly reduces the risk of non-specific amplification, as a lower melting temperature can be used across the PCR cycling protocol, but will accommodate all targets. In this way, a future assay may be designed to incorporate the novel pan-*Leishmania* assay to screen a given sample, then a reflex assay may be used to identify the causative *Leishmania* species. In intercalating self-quenching probes, such as those used in this assay, the fluorescent dye and quencher are at separate ends, that are in a hairpin conformation when not bound to target [21]. This gives less non-specific fluorescence, as the probe is in close proximity to the quencher, and, thus, is more effectively quenched.(See Supplementary Materials)

*Leishmania* DNA-based detection in the laboratory is dominated by cPCR, nested PCR or qPCR. Conventional PCR has sensitivities ranging from 56% to 100%, depending on clinical specimen and gene target [8,22]. In nested PCR, an inner and outer set of primers are designed and tested in two rounds to increase sensitivity and specificity [23]. In an Iranian study of cutaneous leishmaniasis patients, it gave a sensitivity of 100% [24]. Both these methods, however, are time-consuming and laborious, requiring gel electrophoresis and a transilluminator for imaging post-PCR. This may also leave the laboratory open to contamination risk during these post-PCR methods. Quantitative PCR is a closed-tube system, where one step is required between DNA addition and result, and results may be read in real-time [25]. It achieves sensitivities and specificities of up to 100% [26,27]. The novel qPCR achieved a lower LOD than cPCR, an outcome seen in other *Leishmania* assays utilising various targets [14,28,29]. Our future studies will determine the clinical sensitivity of samples previously tested positive for visceral leishmaniasis, to complement the clinical data obtained here.

Currently, there are very few commercial assays available on the market for the detection of all *Leishmania* species, particularly those based on the detection of *Leishmania* DNA, however, no formal evaluations are described in the scientific literature. Primer Design provide a primer and probe set with mastermix and controls, which claims to detect all *Leishmania* species, based on the cytochrome b gene. This is a qPCR test, providing lyophilized components, with a sensitivity of 100 copies (http://www.genesig.com/assets/files/leishmania_spp_std.pdf). Another assay detects *L. major* only (MyBioSource), through a qPCR assay containing the primers, probes, mastermix and controls. It claims a sensitivity of 100 copies of target template (https://www.mybiosource.com/images/tds/protocol_manuals/000000-799999/MBS486088_Easy.pdf). BioKits have a cPCR kit detection *Leishmania* spp., containing ready-to-use PCR mix and positive control, with a sensitivity of 20 copies/mL (http://www.biokits.com/productinfo/3587/Leishmania-sp.-PCR-Detection-Kit.html). The US army has an FDA-approved *Leishmania* qPCR detection kit called SMART Leish, developed in conjunction with Cepheid and the Walter Reed Army Institute of Research for the diagnosis of species associated with cutaneous leishmaniasis, with an LOD of four genome copies (http:/www.accessdata.fda.gov/cdrh_docs/pdf8/K081868.pdf). Its use is restricted to the Department of Defense laboratories, and thus not available commercially. 

The novel pan-*Leishmania* assay provides a simple, economical solution for a high-tech molecular detection system, while retaining excellent sensitivity and specificity, that can be easily used in reference and satellite laboratories alike. Moreover, the automated nature of this system and its low cost means its application is feasible in many countries where leishmaniasis is endemic, which may lack the finances and expertise to implement high-tech laboratory diagnostics, such as qPCR. Such an efficient workflow and quality performance assures that reliable patient results can be diagnosed quickly, treatment regimes can be administered, and prognosis can be assessed.

## Figures and Tables

**Table 1:** List of organisms used in this study for cross-reactivity testing for the novel bisulphite conversion assay.

Specimen Number	Organism
1	*Acinetobacter baumanni*
2	*Bacillus cereus*
3	*Bacillus subtilis*
4	*Clostridium perfringens*
5	*Clostridium sordelli*
6	*Escherichia coli*
7	*Haemophilus influenzae*
8	*Klebsiella oxytoca*
9	*Klebsiella pneumoniae*
10	*Moraxella cattaharalis*
11	*Proteus mirabilis*
12	*Proteus vulgaris*
13	*Pseudomonas aeruginosa*
14	*Staphylococcus aureus*
15	*Staphylococcus hominis*
16	*Streptococcus pyogenes*
17	*Streptococcus sp. (mutans)*
18	*Yersinia sp.*
19	*Mycobacteria abscessus*
20	*Mycobacteria marinum*
21	*Mycobacteria sp.*
22	Herpes Simplex Virus Type I
23	Herpes Simplex Virus Type II
24	Varicella Zoster Virus
25	*Trichophyton tonsurans*
26	*Trichophyton mentagrophytes*
27	*Microsporum canis*
28	*Aspergillus fumigatus*
29	*Acromium pulluans*
30	*Acromium strictum*
31	*Aspergillus sp.*
32	*Bipolaris sp.*
33	*Fusarium sp.*
34	*Penicillium sp.*
35	*Scedosporium prolificans*
36	*Trichophyton rubrum*
37	Bovine
38	Human
39	*Trypanosoma cruzi*
40	*Crithidia lucilae*
41	*Trichomonas vaginalis*
42	*Giardia intestinalis*
43	*Entamoeba histolytica*

**Table 2:** Detection limit of the conventional and novel PCR assays.

Species	Supplier	Schönian Method	Novel Method
*L. donovani*	ATCC	100 cells/PCR	10 cells/PCR
*L. braziliensis*	ATCC	100 cells/PCR	10 cells/PCR
*L. tropica*	ATCC	100 cells/PCR	10 cells/PCR
*L. amazonensis*	ATCC	100 cells/PCR	10 cells/PCR
*L. mexicana*	ATCC	100 cells/PCR	100 cells/PCR
*L. major*	ATCC	10 cells/PCR	10 cells/PCR
*L. infantum*	Vircell	1000 copies/PCR	10 copies/PCR

**Table 3:** Summary of the Observed Precision Estimates for the novel assay.

*Leishmania* Species and Copy Number	Mean (Ct)	SD (Ct)	CV (%)
*L. donovani* 1000 c/PCR	30.26	0.88	2.92
*L. donovani* 100 c/PCR	33.28	0.99	2.98
*L. braziliensis* 100 c/PCR	30.78	0.87	2.84
*L. braziliensis* 10 c/PCR	33.71	0.67	1.99
*L. tropica* 100 c/PCR	34.07	0.95	2.80
*L. tropica* 10 c/PCR^1^	37.83	2.31	6.11
*L. amazonensis* 100 c/PCR	31.29	1.09	3.50
*L. amazonensis* 10 c/PCR	34.24	2.19	6.39
*L. mexicana* 1000 c/PCR	33.08	1.19	3.61
*L. mexicana* 100 c/PCR	36.22	1.52	4.19
*L. major* 100 c/PCR	31.96	0.84	2.62
*L. major* 10 c/PCR	35.09	0.74	2.10
*L. infantum* 1000 c/PCR	29.44	1.22	4.13
*L. infantum* 100 c/PCR	32.23	1.47	4.57

^1^ For *L. tropica*, only 2/3 replicates were achieved on four of the five consecutive days tested. In order to assess mean, SD and CV, the negative data points were excluded from the data set.

## Supplementary Materials

The following are available online at https://www.mdpi.com/2414-6366/4/4/135/s1, Figure S1: Conventional and bisulphite converted alignments for the 18S rDNA gene, Table S1: Conventional and bisulphite converted primer and probe designs for the novel assay.
