# Supplementary material for: Semi-Quantitative, Duplexed qPCR Assay for the Detection of Leishmania spp. Using Bisulphite Conversion Technology
Source: Trop Med Infect Dis. 2019 Nov 1;4(4):135. doi: 10.3390/tropicalmed4040135 (PMC6958480; doi:10.3390/tropicalmed4040135)
Supplement: Supplementary file 1 [file tropicalmed-04-00135-s001.pdf]

Conventional sequences

Leishmania colombiensis	AGGCTACCGTTTCGGCTTTTGTTGGTTTTAAAGGTCTATTGGAGATTATGGAGCTGTGCGACAAGCGCTTTCCCATCGCAACTCCGGTTCGGTGTGTG
Leishmania naiffi	AGGCTACCGTTTCGGCTTTTGTTGGTTTTAAAGGTCTATTGGAGATTATGGAGCTGTGCGACAAGCGCTCTCCCATCGCAACTTCGGTTCGGTGTGTG
Leishmania panamensis	AGGCTACCGTTTCGGCTTTTGTTGGTTTTAAAGGTCTATTGGAGATTATGGAGCTGTGCGACAAGCGCTCTCCCATCGCAACTTCGGTTCGGTGTGTG
Leishmania guyanensis	AGGCTACCGTTTCGGCTTTTGTTGGTTTTAAAGGTCTATTGGAGATTATGGAGCTGTGCGACAAGCGCTCTCCCATCGCAACTTCGGTTCGGTGTGTG
Leishmania braziliensis	AGGCTACCGTTTCGGCTTTTGTTGGTTTTAAAGGTCTATTGGAGATTATGGAGCTGTGCGACAAGCGCTCTCCCATCGCAACTTCGGTTCGGTGTGTG
Leishmania lainsoni	AGGCTACCGTTTCGGCTTTTGTTGGTTTTAAAGGTCTATTGGAGATTATGGAGCTGTGCGACAAGCGCTCTCCCATCGCAACTTCGGTTCGGTGTGTG
Leishmania shawi	AGGCTACCGTTTCGGCTTTTGTTGGTTTTAAAGGTCTATTGGAGATTATGGAGCTGTGCGACAAGCGCTCTCCCATCGCAACTTCGGTTCGGTGTGTG
Leishmania amazonensis	AGGCTACCGTTTCGGCTTTTGTTGGTTTTAAAGGTCTATTGGAGATTATGGAGCTGTGCGACAAGTGCTTTCCCATCGCAACTTCGGTTCGGTGTGTG
Leishmania mexicana	AGGCTACCGTTTCGGCTTTTGTTGGTTTTAAAGGTCTATTGGAGATTATGGAGCTGTGCGACAAGTGCTTTCCCATCGCAACTTCGGTTCGGTGTGTG
Leishmania aethiopica	AGGCTACCGTTTCGGCTTTTGTTGGTTTTAAAGGTCTATTGGAGATTATGGAGCTGTGCGACAAGTGCTTTCCCATCGCAACTTCGGTTCGGTGTGTG
Leishmania donovani	AGGCTACCGTTTCGGCTTTTGTTGGTTTTAAAGGTCTATTGGAGATTATGGAGCTGTGCGACAAGTGCTTTCCCATCGCAACCTCGGTTCGGTGTGTG
Leishmania infantum	AGGCTACCGTTTCGGCTTTTGTTGGTTTTAAAGGTCTATTGGAGATTATGGAGCTGTGCGACAAGTGCTTTCCCATCGCAACCTCGGTTCGGTGTGTG
Leishmania major	AGGCTACCGTTTCGGCTTTTGTTGGTTTTAAAGGTCTATTGGAGATTATGGAGCTGTGCGACAAGTGCTTTCCCATCGCAACTTCGGTTCGGTGTGTG
Leishmania tropica	AGGCTACCGTTTCGGCTTTTGTTGGTTTTAAAGGTCTATTGGAGATTATGGAGCTGTGCGACAAGTGCTTTCCCATCGCAACTTCGGTTCGGTGTGTG

Bisulphite converted sequences

Leishmania colombiensis	AGGTTATTGTTTTGGTTTTTGTTGGTTTTAAAGGTTTATTGGAGATTATGGAGTTGTGTGATAAGTGTTTTTTTATTGTAATTTTGGTTTGGTGTGTG
Leishmania naiffi	AGGTTATTGTTTTGGTTTTTGTTGGTTTTAAAGGTTTATTGGAGATTATGGAGTTGTGTGATAAGTGTTTTTTTATTGTAATTTTGGTTTGGTGTGTG
Leishmania panamensis	AGGTTATTGTTTTGGTTTTTGTTGGTTTTAAAGGTTTATTGGAGATTATGGAGTTGTGTGATAAGTGTTTTTTTATTGTAATTTTGGTTTGGTGTGTG
Leishmania guyanensis	AGGTTATTGTTTTGGTTTTTGTTGGTTTTAAAGGTTTATTGGAGATTATGGAGTTGTGTGATAAGTGTTTTTTTATTGTAATTTTGGTTTGGTGTGTG
Leishmania braziliensis	AGGTTATTGTTTTGGTTTTTGTTGGTTTTAAAGGTTTATTGGAGATTATGGAGTTGTGTGATAAGTGTTTTTTTATTGTAATTTTGGTTTGGTGTGTG
Leishmania lainsoni	AGGTTATTGTTTTGGTTTTTGTTGGTTTTAAAGGTTTATTGGAGATTATGGAGTTGTGTGATAAGTGTTTTTTTATTGTAATTTTGGTTTGGTGTGTG
Leishmania shawi	AGGTTATTGTTTTGGTTTTTGTTGGTTTTAAAGGTTTATTGGAGATTATGGAGTTGTGTGATAAGTGTTTTTTTATTGTAATTTTGGTTTGGTGTGTG
Leishmania amazonensis	AGGTTATTGTTTTGGTTTTTGTTGGTTTTAAAGGTTTATTGGAGATTATGGAGTTGTGTGATAAGTGTTTTTTTATTGTAATTTTGGTTTGGTGTGTG
Leishmania mexicana	AGGTTATTGTTTTGGTTTTTGTTGGTTTTAAAGGTTTATTGGAGATTATGGAGTTGTGTGATAAGTGTTTTTTTATTGTAATTTTGGTTTGGTGTGTG
Leishmania aethiopica	AGGTTATTGTTTTGGTTTTTGTTGGTTTTAAAGGTTTATTGGAGATTATGGAGTTGTGTGATAAGTGTTTTTTTATTGTAATTTTGGTTTGGTGTGTG
Leishmania donovani	AGGTTATTGTTTTGGTTTTTGTTGGTTTTAAAGGTTTATTGGAGATTATGGAGTTGTGTGATAAGTGTTTTTTTATTGTAATTTTGGTTTGGTGTGTG
Leishmania infantum	AGGTTATTGTTTTGGTTTTTGTTGGTTTTAAAGGTTTATTGGAGATTATGGAGTTGTGTGATAAGTGTTTTTTTATTGTAATTTTGGTTTGGTGTGTG
Leishmania major	AGGTTATTGTTTTGGTTTTTGTTGGTTTTAAAGGTTTATTGGAGATTATGGAGTTGTGTGATAAGTGTTTTTTTATTGTAATTTTGGTTTGGTGTGTG
Leishmania tropica	AGGTTATTGTTTTGGTTTTTGTTGGTTTTAAAGGTTTATTGGAGATTATGGAGTTGTGTGATAAGTGTTTTTTTATTGTAATTTTGGTTTGGTGTGTG

Figure S1. Conventional and bisulphite converted alignments for the 18S rDNA gene.

Table 1. Conventional and bisulphite converted primer and probe designs for the novel assay.

	Conventional Sequence	Tm	Bisulphite Converted Sequence	Tm
Forward Primer	CTACCGTTTCGGCTTTTG	60.9	TTATTGTTTTGGTTTTTG	50.8
Reverse Primer	TCCCATCGCAACCTCGGTTC	71.6	TTTTATTGTAATTTTGGTTT	50.8
Probe	GGAGATTATGGAGCTGTGCGACA	69.3	GGAGATTATGGAGTTGTGTGATA	59.4

>gi|261499643|gb|GQ920678.1| Leishmania aethiopica isolate 1214 18S ribosomal RNA gene, nete sequence
>gi|1302626|gb|U21687.1|LAU21687 Leishmania amazonensis 18S ribosomal RNA, intergenic region, promoter region, and external transcribed spacer, partial sequence

>gi|577861717|emb|HG512966.1| Leishmania braziliensis genomic DNA containing 18S rRNA gene, ITS1 and 5.8S rRNA gene, strain MHOM/PE/03/LH2511
>gi|656462896|gb|KF302750.1| Leishmania colombiensis isolate IGOM/PA/1985/E582.34 18S ribosomal RNA gene, partial sequence
>gi|79677117|emb|AJ634376.1| Leishmania donovani 18S rRNA gene (partial), 5.8S rRNA gene, 28S rRNA gene (partial), ITS1 and ITS2, isolate MHOM/IN/00/DEVI
>gi|254847849|gb|GQ332358.1| Leishmania guyanensis 18S ribosomal RNA gene, complete sequence; and internal transcribed spacer 1, partial sequence
>gi|891862543|ref|XR_001203206.1| Leishmania infantum JPCM5 18S ribosomal RNA (SSU) RNA rRNA
>gi|575527028|gb|KF041805.1| Leishmania lainsoni isolate IOCL 1023 18S ribosomal RNA gene, partial sequence
>gi|577861714|emb|HG512963.1| Leishmania major genomic DNA containing 18S rRNA gene, ITS1 and 5.8S rRNA gene, strain MHOM/BF/2004/REN04-8 (LEM4886)
>gi|577861716|emb|HG512965.1| Leishmania mexicana genomic DNA containing 18S rRNA gene, ITS1 and 5.8S rRNA gene, strain MHOM/PE/02/LH2312
>gi|575527030|gb|KF041807.1| Leishmania naiffi isolate IOCL 1365 18S ribosomal RNA gene, partial sequence
>gi|254847853|gb|GQ332362.1| Leishmania panamensis 18S ribosomal RNA gene, complete sequence; and internal transcribed spacer 1, partial sequence
>gi|575527031|gb|KF041808.1| Leishmania shawi isolate IOCL 1545 18S ribosomal RNA gene, partial sequence
>gi|577861678|emb|HG512927.1| Leishmania tropica genomic DNA containing 18S rRNA gene, ITS1 and 5.8S rRNA gene, strain MHOM/EG/90/LPN65 (LEM2001)
